# Association of a Fixed 1 mg Dose of Granisetron for Preventing Postoperative Nausea and Vomiting (PONV) in Patients Undergoing Laparoscopic Colorectal Surgery With Multiple PONV Risk Factors: A Retrospective Observational Study

**DOI:** 10.7759/cureus.104701

**Published:** 2026-03-05

**Authors:** Takayuki Morimoto, Kentaro Hara, Akihisa Nishigaki, Keiko Tashiro, Akiha Nakao, Kyoko Nagaoka, Michiko Yamaguchi, Tetsuya Hara

**Affiliations:** 1 Department of Anesthesiology, National Hospital Organization (NHO) Nagasaki Medical Center, Nagasaki, JPN; 2 Department of Anesthesiology and Intensive Care Medicine, Nagasaki University Graduate School of Biomedical Sciences, Nagasaki, JPN; 3 Department of Fundamental Nursing, Faculty of Life Sciences, Kumamoto University, Kumamoto, JPN; 4 Clinical Research Center, National Hospital Organization (NHO) Nagasaki Medical Center, Nagasaki, JPN; 5 Healthcare Management Research Center, Chiba University, Chiba, JPN

**Keywords:** fentanyl, fixed dose, granisetron, laparoscopic colorectal surgery, ponv prophylaxis, postoperative nausea and vomiting (ponv)

## Abstract

Introduction

Granisetron is a selective 5-hydroxytryptamine type 3 (5-HT3) receptor antagonist approved in Japan for the prophylaxis of postoperative nausea and vomiting (PONV). According to the Japanese package insert, a fixed intravenous dose of 1 mg is recommended; however, evidence for its effectiveness in patients undergoing laparoscopic colorectal surgery in a clinical context characterized by multiple established PONV risk factors remains limited. We evaluated the association between prophylactic granisetron 1 mg and PONV-related outcomes in this setting.

Methods

This single-center retrospective observational study included adult patients who underwent elective laparoscopic colorectal surgery between January 2018 and July 2024. All patients received inhalational anesthesia and postoperative analgesia consisting of a peripheral nerve block and continuous intravenous fentanyl infusion. Patients were divided into a granisetron group (G group: 1 mg intravenously at the end of surgery) and a no-granisetron group (N group). The primary outcome was PONV within 24 hours. Secondary outcomes were rescue antiemetic use within 24 hours and complete response (no PONV and no rescue antiemetic use). Multivariable logistic regression was performed with adjustment for prespecified covariates and surgical year. Sensitivity analyses included propensity score overlap weighting and restriction to surgeries performed in 2022 or later. Within the granisetron group, an exploratory multivariable analysis for complete response included sex, age, and granisetron dose per body weight.

Results

A total of 377 patients were included (G group, n = 101; N group, n = 276). PONV occurred less frequently in the granisetron group than in the no-granisetron group (34/101 (33.7%) vs 129/276 (46.7%), p = 0.026), whereas rescue antiemetic use was similar (33/101 (32.7%) vs 96/276 (34.8%), p = 0.806). Complete response tended to be higher in the granisetron group (65/101 (64.4%) vs 146/276 (52.9%), p = 0.061). After adjustment, granisetron was associated with lower odds of PONV (adjusted odds ratio (aOR) 0.355, 95% CI 0.181-0.697; p = 0.003) and higher odds of complete response (aOR 2.826, 95% CI 1.444-5.531; p = 0.002). The association with rescue antiemetic use did not reach statistical significance. Sensitivity analyses using propensity score overlap weighting and restriction to surgeries performed in 2022 or later yielded directionally consistent results, although estimates were less precise.

Conclusions

In patients undergoing laparoscopic colorectal surgery in a setting with multiple established risk factors for PONV, prophylactic administration of a fixed 1 mg dose of granisetron may be associated with improved PONV outcomes within 24 hours.

## Introduction

Postoperative nausea and vomiting (PONV) is one of the most frequent and distressing complications following general anesthesia, occurring in approximately 30% of all surgical patients and up to 80% of patients with multiple risk factors [1,2]. In addition to patient discomfort and dissatisfaction, PONV is associated with delayed recovery, prolonged hospitalization, and increased healthcare costs [3]. In gastrointestinal surgery, PONV may also contribute to serious postoperative complications, including aspiration pneumonia, and may raise concern for anastomotic integrity by increasing intra-abdominal pressure during retching and vomiting [4,5]. Therefore, effective prophylaxis against PONV is especially important in this population.

Laparoscopic colorectal surgery often combines several established risk factors for PONV, including laparoscopy itself, prolonged operative duration, maintenance with volatile anesthetics, and postoperative opioid administration for analgesia [1].

Granisetron is a selective 5-HT3 receptor antagonist that was approved in Japan for PONV prophylaxis in 2021. Current international guidelines recommend multimodal antiemetic prophylaxis for patients at moderate to high risk of PONV [1,2]; however, granisetron is considered effective as a single agent [6]. In Japan, prophylactic options with an explicit on-label indication for PONV are limited, and 5-HT3 receptor antagonists, including granisetron, are among the few available agents under the national health insurance system. According to the package insert in Japan, a fixed intravenous dose of 1 mg is recommended for PONV prophylaxis without weight-based dose adjustment, whereas weight-based dosing (40 μg/kg) is recommended for chemotherapy- or radiotherapy-induced nausea and vomiting [7].

To date, evidence specifically addressing the effectiveness of a fixed 1 mg dose of granisetron in patients undergoing laparoscopic colorectal surgery with multiple PONV risk factors, including volatile anesthesia, prolonged operative duration, and postoperative opioid-based analgesia, remains limited. Consequently, whether this regimen provides sufficient prophylaxis in such patients remains uncertain.

Therefore, we conducted a retrospective observational cohort study to evaluate the effectiveness of the Japan-approved fixed 1 mg regimen of granisetron for PONV prophylaxis in patients undergoing laparoscopic colorectal surgery in a setting with multiple PONV risk factors. Specifically, we compared patients who received prophylactic granisetron 1 mg with those who did not and evaluated the incidence of PONV within the first 24 postoperative hours as the primary outcome. Secondary outcomes included rescue antiemetic use and complete response. We hypothesized that prophylactic granisetron 1 mg would reduce the incidence of PONV within the first 24 postoperative hours compared with no granisetron.

## Materials and methods

Study design and ethics

This single-center retrospective observational study was conducted at NHO Nagasaki Medical Center. The study protocol was approved by the institutional ethics committee (approval number: 2024059), and the requirement for informed consent was waived because of the retrospective design. The study is reported in accordance with the Strengthening the Reporting of Observational Studies in Epidemiology (STROBE) statement [8].

Patient selection

**Figure 1:**
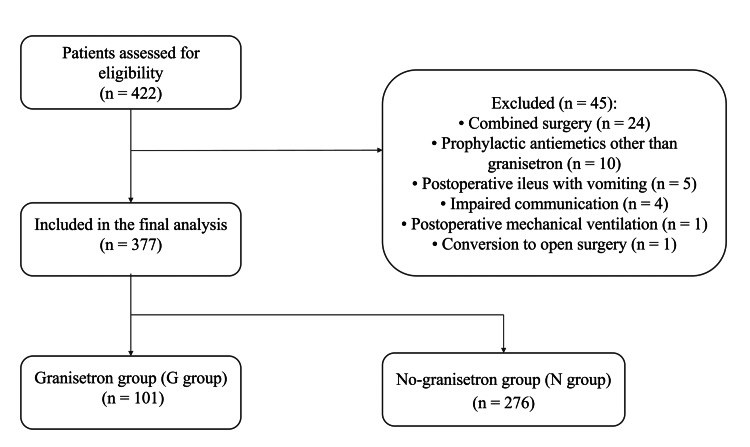
Flow diagram of patient selection. Of the 422 patients assessed for eligibility, 45 were excluded due to combined surgery involving other organs (n = 24), prophylactic use of antiemetics other than granisetron (n = 10), postoperative ileus with vomiting (n = 5), impaired communication (n = 4), postoperative mechanical ventilation (n = 1), and conversion to open surgery (n = 1). A total of 377 patients were included in the final analysis and were categorized into the granisetron group (G group; 1 mg intravenously at the end of surgery; n = 101) and the no-granisetron group (N group; n = 276).

Patient selection is illustrated in Figure 1. Adult patients who underwent elective laparoscopic colorectal surgery between January 2018 and July 2024 were screened for eligibility. Data were retrospectively abstracted from anesthesia records and postoperative nursing documentation in the electronic medical record. Inclusion criteria were maintenance of anesthesia with inhalational anesthetics and postoperative analgesia consisting of an ultrasound-guided abdominal wall block (e.g., transversus abdominis plane block) using ropivacaine and continuous intravenous fentanyl infusion, typically at 0.01 μg/kg/min (bolus doses were provided at the discretion of the treating team when postoperative pain was inadequately controlled). Exclusion criteria were impaired communication, prophylactic use of any antiemetic medication other than granisetron, combined surgery involving other organs, conversion to open surgery, postoperative mechanical ventilation, and postoperative ileus with vomiting.

For the purpose of this study, we considered this cohort to be in a setting with multiple PONV risk factors because laparoscopic colorectal surgery commonly involves several such risk factors, including laparoscopic manipulation, maintenance of anesthesia with volatile agents, prolonged surgical or anesthetic duration, and postoperative opioid administration for analgesia [2,9]. We selected January 2018 as the start date because our institution implemented a standardized postoperative analgesia pathway incorporating ultrasound-guided abdominal wall block in that year, enabling a more homogeneous analgesic strategy across the study period. Granisetron for PONV prophylaxis became available in Japan in 2021 and was introduced into clinical practice at our institution beginning in 2022. Therefore, to minimize potential selection bias related to clinician-directed prophylaxis after granisetron introduction (i.e., preferential administration to patients perceived to be at higher risk for PONV), we included cases from before 2022 and addressed this concern analytically by adjusting for surgical year in the primary models and performing a sensitivity analysis restricted to surgeries performed in 2022 or later (described below).

Study groups

Patients were categorized into two groups: those who received intravenous granisetron 1 mg intraoperatively (G group) and those who did not receive granisetron (N group). Granisetron administration was determined by the attending anesthesiologist at the end of surgery, reflecting routine clinical practice at the study institution. Because granisetron administration was clinician-directed, we adjusted for clinically relevant covariates in the multivariable analyses.

Outcomes

The primary outcome was PONV within 24 hours after surgery, defined as any documented nausea and/or vomiting episode in the medical record. Secondary outcomes were rescue antiemetic administration (IV metoclopramide 10 mg, clinician-directed per routine postoperative care) within 24 hours after surgery and complete response. Complete response was defined as the absence of both PONV and rescue antiemetic administration within 24 hours after surgery. We selected the first 24 postoperative hours as the observation window because major evidence syntheses summarized in the Guidelines commonly use 24-hour PONV/POV outcomes as primary endpoints [2].

Statistical analysis

Continuous variables are presented as median (interquartile range) and were compared using the Mann-Whitney U test. Categorical variables are presented as numbers (%) and were compared using Fisher’s exact test or the chi-square test. All tests were two-sided, and p-values < 0.05 were considered statistically significant.

For the primary analysis, perioperative variables were compared between the granisetron group (G group) and the no-granisetron group (N group). To adjust for confounding, separate multivariable logistic regression models were fitted for (1) PONV within 24 hours, (2) rescue antiemetic use within 24 hours, and (3) complete response. Covariates were selected a priori based on clinical relevance and availability in the medical record: granisetron administration (yes/no), sex, age, anesthesia time, intraoperative fluid balance, non-smoking status, total intraoperative fentanyl dose, and surgical year. For interpretability, continuous covariates were scaled as follows: anesthesia time per 1 hour, fluid balance per 1 L, total intraoperative fentanyl dose per 100 μg, and surgical year per 1 year. Results are reported as adjusted odds ratios (ORs) with 95% confidence intervals (CIs).

We assessed multicollinearity among covariates using variance inflation factors (VIFs). Linearity of continuous covariates on the logit scale was evaluated using likelihood ratio tests comparing the main model with models incorporating spline terms (df = 3) for each continuous variable.

Missing data were summarized for each variable in Tables 1, 2. Analyses were conducted using available data, and no imputation was performed.

Although granisetron became available for PONV prophylaxis in Japan in 2021, it was introduced into clinical practice at our institution beginning in 2022. Therefore, the surgical year was included in the primary multivariable models. As sensitivity analyses, we (1) conducted propensity score-based inverse probability weighting using overlap weights based on the same prespecified covariates and estimated weighted ORs for the primary outcomes, and (2) repeated the multivariable models restricting the cohort to surgeries performed in 2022 or later to reduce potential calendar-time confounding from time-varying practice changes.

Because this was a retrospective cohort study, the sample size was determined by the number of eligible patients during the study period, and no a priori power calculation was performed.

Within the G group, an exploratory multivariable logistic regression analysis was performed with complete response as the dependent variable to identify factors associated with failure of prophylaxis. Covariates included sex, age, and granisetron dose per body weight (mg/kg).

Statistical analyses were performed using EZR (Saitama Medical Center, Jichi Medical University, Saitama, Japan).

## Results

A total of 422 patients met the initial inclusion criteria. After exclusion of 45 patients, 377 patients were included in the final analysis (Figure 1). These patients were divided into the G group (n = 101) and the N group (n = 276).

Baseline patient characteristics are summarized in Table 1. The proportion of female patients was significantly higher in the G group than in the N group (58.4% vs 38.4%, p < 0.001). There were no significant differences between the groups with respect to age, ASA physical status, body mass index, or smoking status. The duration of anesthesia and surgery was significantly shorter in the G group than in the N group, whereas total intraoperative fentanyl dose, fluid balance, anesthetic agents used, and the use of non-opioid analgesics did not differ significantly between the groups. A full Apfel score [2] could not be calculated because prior PONV or motion sickness history was not consistently available. However, the available components (female sex and non-smoking status) are shown in Table 1, and all patients received postoperative opioids. There were no missing values for the primary outcome or prespecified covariates used in the multivariable models; therefore, the primary analyses included the full cohort. A small number of values were missing for selected postoperative variables and are reported in Table 2.

**Table 1:** Patient characteristics. Values are presented as median (interquartile range) or number (%). NA, not applicable; —, not calculated. Continuous variables were compared using the Mann–Whitney U test. Categorical variables were compared using Fisher’s exact test or the chi-square test. For Fisher’s exact test, odds ratios (G group vs N group) with 95% confidence intervals are reported; for the Mann–Whitney U and chi-square tests, Z and χ² values are reported, respectively. Missing indicates unavailable data in the medical record. ^†^Fisher’s exact test. ^‡^Chi-square test. PONV, postoperative nausea and vomiting; ASA-PS, American Society of Anesthesiologists physical status; BMI, body mass index.

Variables	G group (n=101)	N group (n=276)	Statistic	p value	Missing G group	Missing N group
Female sex, n (%)^†^	59 (58.4)	106 (38.4)	2.253 (1.416-3.584)	<0.001	0	0
ASA-PS^‡^			0.212	0.900	0	0
I, n (%)	6 (5.9)	20 (7.2)	—	—	0	0
II, n (%)	77 (76.2)	206 (74.6)	—	—	0	0
III, n (%)	18 (17.8)	50 (18.1)	—	—	0	0
Age, years	70 (60-77)	71 (63-79)	-0.610	0.542	0	0
Height, cm	158.8 (151.4-163.9)	161.1 (152.5-168.0)	-1.759	0.079	0	0
Weight, kg	54.6 (47.6-63.0)	57.4 (49.9-67.7)	-1.326	0.185	0	0
BMI, kg/m²	22.3 (19.4-26.0)	22.8 (20.0-24.7)	-0.345	0.730	0	0
Non-smoker, n (%)^†^	82 (81.2)	221 (80.0)	1.074 (0.601-1.918)	0.884	0	0
Duration of anesthesia, min	362 (289-449)	412 (339-471)	-3.395	0.001	0	0
Duration of surgery, min	280 (208-360)	322 (248-380)	-2.826	0.005	0	0
Estimated blood loss, mL	15 (5-40)	20 (10-50)	-1.529	0.126	0	0
Fluid balance, mL	1848 (1452-2138)	1986 (1476-2473)	-1.865	0.062	0	0
Anesthetic agent^†^			1.411 (0.799-2.493)	0.271	0	0
sevoflurane, n (%)	82 (81.2)	208 (75.4)	—	—	0	0
desflurane, n (%)	19 (18.8)	68 (24.6)	—	—	0	0
Total intraoperative fentanyl dose, μg	280 (150-430)	246 (147-426)	0.446	0.656	0	0
Granisetron dose per body weight, μg/kg	18.3 (15.8-21.0)	NA	—	—	0	NA
Acetaminophen use, n (%)^†^	96 (95.0)	250 (90.6)	1.997 (0.745-5.350)	0.206	0	0
Flurbiprofen use, n (%)^†^	70 (69.3)	205 (74.3)	0.782 (0.474-1.292)	0.360	0	0

Postoperative outcomes are presented in Table 2. The incidence of PONV was lower in the G group than in the N group (33.7% vs 46.7%, p = 0.026). The unadjusted absolute risk reduction in PONV was 13.1% (95% CI 2.1%-24.0%). The proportion of patients receiving rescue antiemetics did not differ significantly between groups (32.7% vs 34.8%, p = 0.806). Vomiting was numerically lower in the granisetron group (13.9% vs 22.5%, p = 0.081). Complete response occurred in 64.4% of patients in the granisetron group and 52.9% of patients in the no-granisetron group (p = 0.061). Missing data are reported in Table 2.

**Table 2:** Postoperative outcomes. Values are presented as median (interquartile range) or number (%). Continuous variables were compared using the Mann–Whitney U test. Categorical variables were compared using Fisher’s exact test. For Fisher’s exact test, odds ratios (G group vs N group) with 95% confidence intervals are reported; Z values are reported for the Mann–Whitney U test. Missing indicates unavailable data among applicable cases. Rescue antiemetic: intravenous metoclopramide 10 mg administered within 24 hours after surgery. Complete response: The absence of both PONV and rescue antiemetic administration within 24 hours after surgery. ^†^Fisher’s exact test. PONV, postoperative nausea and vomiting.

Variables	G group (n=101)	N group (n=276)	Statistic	p value	Missing G group	Missing N group
PONV within 24 h, n (%)^†^	34 (33.7)	129 (46.7)	0.578 (0.359–0.930)	0.026	0	0
Rescue antiemetic within 24 h, n (%)^†^	33 (32.7)	96 (34.8)	0.910 (0.561–1.476)	0.806	0	0
Number of rescue antiemetic doses	1 (1–2) (n=32)	1 (1–2) (n=95)	0.000	1.000	1	1
Vomiting within 24 h, n (%)^†^	14 (13.9)	62 (22.5)	0.555 (0.295–1.044)	0.081	0	0
Additional fentanyl bolus, n (%)^†^	26 (25.7)	58 (21.0)	1.303 (0.766–2.218)	0.331	0	0
Complete response, n (%)^†^	65 (64.4)	146 (52.9)	1.608 (1.004–2.574)	0.061	0	0
Number of fentanyl boluses	2 (1–2) (n=26)	1 (1–2) (n=58)	1.037	0.300	0	0
Reduction of fentanyl infusion, n (%)^†^	25 (24.8)	75 (27.2)	0.882 (0.522–1.489)	0.694	0	0
Discontinuation of fentanyl infusion, n (%)^†^	3 (3.0)	11 (4.0)	0.737 (0.201–2.699)	0.768	0	0
Oral rehydration intake, %	50 (50–100)	50 (50–100)	-1.453	0.146	5	13
Postoperative day of diet initiation	4 (4–6)	4 (4–6)	-1.331	0.183	0	2
Length of hospital stay, days	10 (9–14)	11 (9–14)	-0.574	0.566	0	0
Aspiration pneumonia, n (%)^†^	1 (1.0)	5 (1.0)	0.542 (0.063–4.696)	1.000	0	0

Multivariable logistic regression results are presented in Table 3. No problematic multicollinearity was identified (all VIFs < 2.0), and no evidence of non-linearity on the logit scale was observed for continuous covariates (all likelihood ratio tests p > 0.05). After adjustment for prespecified covariates and surgical year, granisetron was independently associated with lower odds of PONV (adjusted OR 0.355, 95% CI 0.181-0.697; p = 0.003) and higher odds of complete response (adjusted OR 2.826, 95% CI 1.444-5.531; p = 0.002). For rescue antiemetic use, granisetron showed a nonsignificant trend toward lower odds (adjusted OR 0.516, 95% CI 0.260-1.021; p = 0.057). Female sex was associated with higher odds of both PONV and rescue antiemetic use and with lower odds of complete response, while non-smoking status was associated with higher odds of PONV and lower odds of complete response. Anesthesia time was not independently associated with PONV (aOR 1.018, 95% CI 0.872-1.188; p = 0.823), suggesting that the between-group difference in procedural duration was unlikely to fully explain the observed association.

**Table 3:** Multivariable logistic regression for PONV, rescue antiemetic use, and complete response within 24 hours. Complete response was defined as the absence of both PONV and rescue antiemetic administration within 24 hours after surgery. Scaling: anesthesia time per 1 hour; fluid balance per 1 L; total intraoperative fentanyl dose per 100 μg; surgical year per 1 year. PONV, postoperative nausea and vomiting, aOR, adjusted odds ratio; CI, confidence interval.

Variables	PONV aOR (95% CI)	PONV p value	Rescue antiemetic aOR (95% CI)	Rescue antiemetic p value	Complete response aOR (95% CI)	Complete response p value
Granisetron administration	0.355 (0.181-0.697)	0.003	0.516 (0.260-1.021)	0.057	2.826 (1.444-5.531)	0.002
Female sex	3.082 (1.895-5.010)	<0.001	3.135 (1.908-5.150)	<0.001	0.311 (0.191-0.506)	<0.001
Age	0.999 (0.979-1.019)	0.914	1.004 (0.984-1.026)	0.676	0.998 (0.978-1.018)	0.861
Anesthesia time (per 1 hour)	1.018 (0.872-1.188)	0.823	0.990 (0.845-1.160)	0.901	0.989 (0.848-1.155)	0.893
Fluid balance (per 1 L)	0.722 (0.490-1.066)	0.101	0.880 (0.591-1.310)	0.529	1.448 (0.980-2.140)	0.063
Non-smoking	2.322 (1.206-4.473)	0.012	1.703 (0.855-3.393)	0.130	0.450 (0.235-0.862)	0.016
Total intraoperative fentanyl dose (per 100 μg)	1.090 (0.969-1.226)	0.150	1.060 (0.939-1.196)	0.349	0.891 (0.792-1.002)	0.054
Surgical year (per 1 year)	1.073 (0.892-1.292)	0.456	1.148 (0.946-1.393)	0.162	0.904 (0.751-1.089)	0.288

To address potential confounding by indication suggested by baseline imbalances, we performed a propensity score-based inverse probability weighting sensitivity analysis using overlap weights. Propensity score overlap weighting achieved good covariate balance, with all covariates showing an absolute standardized mean difference (SMD) < 0.10. Overlap-weighted estimates for PONV, rescue antiemetic use, and complete response are shown in Table 4. The overlap-weighted analyses were directionally; however, the estimate for PONV did not reach statistical significance (OR 0.393, 95% CI 0.142-1.087), indicating limited precision and a nonconfirmatory sensitivity result.

**Table 4:** Sensitivity analysis of prophylactic granisetron 1 mg vs no granisetron using propensity score overlap weighting. Complete response was defined as the absence of both PONV and rescue antiemetic administration within 24 hours after surgery. Overlap weights were 1−propensity score (PS) for treated and PS for untreated patients. PONV, postoperative nausea and vomiting; OR, odds ratio; CI, confidence interval.

Variables	OR	95% CI	p value
PONV	0.393	0.142-1.087	0.072
Rescue antiemetic	0.483	0.172-1.356	0.167
Complete response	2.711	0.987-7.448	0.053

In a separate sensitivity analysis restricted to surgeries performed in 2022 or later (n = 197), granisetron remained associated with lower odds of PONV (aOR 0.442, 95% CI 0.208-0.943; p = 0.035) and higher odds of complete response (aOR 2.397, 95% CI 1.123-5.115; p = 0.024), while the association with rescue antiemetic use did not reach statistical significance (aOR 0.510, 95% CI 0.234-1.112; p = 0.091) (Table 5).

**Table 5:** Sensitivity analysis restricted to surgeries performed in 2022 or later. Multivariable logistic regression models were refitted in the cohort restricted to surgeries performed in 2022 or later (n=197). Complete response was defined as the absence of both PONV and rescue antiemetic administration within 24 hours after surgery. Scaling: anesthesia time per 1 hour; fluid balance per 1 L; total intraoperative fentanyl dose per 100 μg; surgical year per 1 year. PONV, postoperative nausea and vomiting, aOR, adjusted odds ratio; CI, confidence interval.

Predictor	PONV aOR (95% CI)	p	Rescue antiemetic aOR (95% CI)	p	Complete response aOR (95% CI)	p
Granisetron (yes vs no)	0.442 (0.208-0.943)	0.035	0.510 (0.234-1.112)	0.091	2.397 (1.123-5.115)	0.024
Female sex	2.693 (1.306-5.557)	0.007	3.517 (1.647-7.513)	0.001	0.328 (0.158-0.680)	0.003
Age (per 1 year)	1.001 (0.975-1.029)	0.924	1.003 (0.976-1.031)	0.818	0.994 (0.968-1.021)	0.658
Anesthesia time (per 1 hour)	1.018 (0.816-1.269)	0.877	1.022 (0.817-1.279)	0.847	1.006 (0.807-1.254)	0.958
Fluid balance (per 1 L)	0.618 (0.350-1.090)	0.097	0.667 (0.375-1.187)	0.169	1.773 (1.000-3.143)	0.050
Non-smoking	1.281 (0.548-2.998)	0.568	1.095 (0.453-2.647)	0.841	0.819 (0.352-1.908)	0.644
Total intraoperative fentanyl dose (per 100 μg)	1.051 (0.879-1.257)	0.585	1.116 (0.930-1.340)	0.239	0.890 (0.745-1.064)	0.201
Surgical year (per 1 year)	1.222 (0.796-1.875)	0.359	1.346 (0.863-2.101)	0.190	0.783 (0.510-1.203)	0.264

In an exploratory analysis within the granisetron group (n = 101), female sex was associated with a lower likelihood of complete response, while granisetron dose per body weight was not significantly associated with complete response (Table 6).

**Table 6:** Exploratory multivariable logistic regression for complete response within the Granisetron group (n=101). Complete response was defined as the absence of both PONV and rescue antiemetic administration within 24 hours after surgery. Granisetron dose per body weight was calculated as 1 mg divided by body weight (kg). PONV, postoperative nausea and vomiting; aOR, adjusted odds ratio; CI, confidence interval.

Variables	aOR (95% CI)	p value
Female sex	0.270 (0.097–0.751)	0.012
Age	0.966 (0.931–1.003)	0.074
Granisetron dose per body weight (per 0.01 mg/kg)	1.799 (0.525–6.168)	0.350

## Discussion

In this retrospective observational study of patients undergoing laparoscopic colorectal surgery in a setting with multiple PONV risk factors, prophylactic administration of a fixed 1 mg dose of granisetron was associated with a lower incidence of PONV and higher odds of complete response, while the association with rescue antiemetic use did not reach statistical significance. To our knowledge, no prior studies have evaluated a fixed 1 mg granisetron regimen specifically in laparoscopic colorectal surgery in a setting characterized by multiple PONV risk factors, including volatile anesthesia, prolonged operative duration, and postoperative opioid-based analgesia.

The incidence of PONV has been reported to reach up to 80% among patients with multiple risk factors [1,2]. In laparoscopic surgery studies using granisetron for PONV prophylaxis, the reported incidence of PONV generally ranged from 16.3% to 48.0% with fixed-dose regimens (1-3 mg) [10-15] and from 6.0% to 23.0% with weight-based regimens (40-50 μg/kg) [16,17]. These values are presented descriptively for context and should not be interpreted as evidence of a dose-response relationship, given substantial heterogeneity in patient characteristics, surgical procedures, and baseline PONV risk. In our cohort of patients undergoing laparoscopic colorectal surgery with volatile anesthesia, prolonged operative duration, and postoperative opioid-based analgesia, prophylactic granisetron 1 mg was associated with a lower incidence of PONV (33.7%), which was within the range reported in prior laparoscopic surgery studies. Notably, although some prior laparoscopic studies included a 1 mg dose, none specifically focused on colorectal surgery combined with postoperative opioid-based analgesia under volatile anesthesia with prolonged operative duration. These findings suggest that granisetron 1 mg may provide clinically meaningful prophylaxis against PONV even in clinical contexts characterized by multiple established PONV risk factors.

In contrast to the reduction in PONV, rescue antiemetic use did not differ significantly between groups, and the adjusted association between granisetron and rescue antiemetic use did not reach statistical significance. This apparent discordance is plausible because PONV was defined based on routine clinical documentation and may capture mild nausea that does not necessarily prompt treatment, whereas rescue antiemetic administration reflects clinical thresholds, patient preferences, and local practice patterns [1,18]. Therefore, rescue antiemetic use should be interpreted as a complementary outcome rather than a direct surrogate for symptom burden. We also reported complete response (no PONV and no rescue antiemetic use) as a composite measure of overall prophylaxis success; however, this composite endpoint should be interpreted cautiously because both components may be influenced by documentation and practice patterns.

We next considered the dosage of granisetron. Because all patients in the granisetron group received a fixed 1 mg dose, per-weight exposure varied (median 18.3 μg/kg (IQR 15.8-21.0)). In the exploratory multivariable analysis within the granisetron group using complete response as the dependent variable, granisetron dose per body weight was not significantly associated with prophylaxis success. Previous studies have yielded inconsistent findings regarding the dose-response relationship of granisetron for PONV prophylaxis. Some reports have suggested that higher doses of granisetron are associated with improved prophylactic efficacy against PONV [19,20], whereas others have reported no additional benefit with increasing doses beyond a certain threshold [21-23]. In addition, it has been reported that granisetron doses of 5 μg/kg or higher provide effective PONV prophylaxis, with no further incremental benefit observed at higher doses [24].

However, most dose-ranging studies in PONV have been designed primarily around clinical outcomes and have seldom included concurrent assessment of pharmacokinetic exposure and pharmacodynamic markers, thereby limiting mechanistic interpretation of the observed dose-response relationship. In chemotherapy settings in which pharmacokinetic sampling has been conducted, exposure-response relationships have not identified a clear plasma concentration threshold for antiemetic efficacy [25]. In keeping with this, receptor-occupancy theory-based analyses indicate a plateau in clinical gain after attainment of sufficient 5-HT3 receptor blockade [26]. Notably, receptor occupancy may vary substantially between individuals under the same dosing regimen [27]. Moreover, individualized approaches linking granisetron concentrations to estimates of emetogenic drive suggest that variability in the emetogenic stimulus may modulate efficacy even under similar dosing [28].

Taken together, these observations are consistent with our finding that, within the exposure range achieved by a fixed 1 mg regimen in this cohort, weight-normalized exposure variability was not associated with complete response. Accordingly, dose escalation beyond 1 mg based solely on body weight may provide limited additional benefit. However, because this study did not include patients receiving granisetron doses greater than 1 mg and did not directly compare different dosing regimens, it cannot be concluded that doses exceeding 1 mg are unnecessary. In addition, because we did not measure granisetron concentrations or pharmacodynamic biomarkers in the perioperative setting, we could not directly characterize an exposure-response relationship in this cohort. Further studies are warranted to clarify whether higher doses of granisetron provide additional prophylactic benefit against PONV in high-risk patients.

Female sex remained a strong risk factor. In the multivariable models, female sex was associated with higher odds of PONV and rescue antiemetic use and with lower odds of complete response. Furthermore, in the exploratory analysis within the granisetron group, female sex was associated with a lower likelihood of complete response. These findings suggest that granisetron 1 mg alone may be insufficient for PONV prophylaxis in female patients undergoing laparoscopic colorectal surgery and that additional preventive strategies may be required in this population. As recommended by current guidelines [1], multimodal antiemetic prophylaxis should be considered, and postoperative pain management strategies aimed at reducing PONV risk are also important.

In the present study, all patients received continuous intravenous fentanyl infusion for postoperative analgesia. Additional fentanyl bolus administration due to insufficient analgesia was required in 22% of patients, whereas fentanyl dose reduction or discontinuation due to PONV was required in 27% of patients. Previous studies have demonstrated that, in laparoscopic surgery, scheduled postoperative administration of acetaminophen and nonsteroidal anti-inflammatory drugs, in addition to regional anesthesia, can provide effective analgesia while reducing opioid consumption [29,30]. In patients in a setting with multiple PONV risk factors, these opioid-sparing analgesic strategies may represent viable alternatives to continuous postoperative fentanyl infusion and should be considered as part of a comprehensive approach to PONV prophylaxis.

This study has several limitations. First, this was a single-center retrospective observational study, which may limit the generalizability of the findings. Residual confounding due to unmeasured variables cannot be excluded, particularly patient-level PONV risk factors such as a history of motion sickness or prior PONV. In addition, the significant baseline imbalance in female sex suggests potential confounding by indication (i.e., clinicians may have preferentially administered granisetron to patients perceived to be at higher risk for PONV). To mitigate this, we adjusted for prespecified covariates in multivariable models and additionally performed propensity score overlap weighting. The weighted estimates were directionally consistent but imprecise and therefore should be interpreted as supportive rather than confirmatory. Residual confounding and/or time-varying practice changes may still influence the observed associations.

Second, the assessment of postoperative nausea and vomiting was based on routine clinical documentation in the medical records, primarily recorded by nursing staff, and therefore relied on subjective evaluation rather than standardized scoring systems. The severity of nausea was not quantified, which may have resulted in some degree of misclassification of PONV. In addition, recovery-related outcomes such as PACU length of stay were not available because a PACU is not routinely used at our institution. Patient-reported outcomes (e.g., satisfaction) were also not consistently documented and therefore could not be analyzed in this retrospective dataset.

Third, the study design did not include a comparison of different granisetron dosing regimens, as all patients in the granisetron group received a fixed dose of 1 mg. Therefore, the present findings cannot be used to determine whether higher doses of granisetron would provide additional prophylactic benefit in patients in a setting with multiple PONV risk factors.

Fourth, the G group size (n = 101) limited the precision of effect estimates. For example, the unadjusted absolute risk reduction in PONV was 13.1% with a wide 95% CI (2.1% to 24.0%), indicating uncertainty in the magnitude of benefit. Because subgroup analyses further reduce the effective sample size and thus yield even less precise estimates, subgroup findings should be interpreted as hypothesis-generating.

Finally, calendar time could confound treatment selection and outcomes because granisetron was introduced at our institution in 2022. Although residual confounding from time-varying practice changes cannot be fully excluded, the direction of associations remained consistent after adjustment for surgical year and in both sensitivity analyses (propensity score overlap weighting and restriction to surgeries performed in 2022 or later), although estimates were less precise in the sensitivity analyses.

## Conclusions

In patients undergoing laparoscopic colorectal surgery in a setting with multiple PONV risk factors, prophylactic administration of a fixed 1 mg dose of granisetron may be associated with improved PONV outcomes within 24 hours.
